# Spectral sensitivity of cone vision in the diurnal murid *Rhabdomys pumilio*

**DOI:** 10.1242/jeb.215368

**Published:** 2020-05-29

**Authors:** Annette E. Allen, Joshua W. Mouland, Jessica Rodgers, Beatriz Baño-Otálora, Ronald H. Douglas, Glen Jeffery, Anthony A. Vugler, Timothy M. Brown, Robert J. Lucas

**Affiliations:** 1 Division of Neuroscience and Experimental Psychology, Faculty of Biology Medicine and Health, University of Manchester, Manchester, M13 9PT, UK; 2 Division of Diabetes, Endocrinology and Gastroenterology, Faculty of Biology Medicine and Health, University of Manchester, Manchester, M13 9PT, UK; 3 Department of Optometry and Visual Science, City, University of London, London, EC1V 0HB, UK; 4 Institute of Ophthalmology, University College London, London, EC1V 9EL, UK

**Keywords:** Diurnality, Photoreceptor, Opsin

## Abstract

An animal's temporal niche – the time of day at which it is active – is known to drive a variety of adaptations in the visual system. These include variations in the topography, spectral sensitivity and density of retinal photoreceptors, and changes in the eye's gross anatomy and spectral transmission characteristics. We have characterised visual spectral sensitivity in the murid rodent *Rhabdomys pumilio* (the four-striped grass mouse), which is in the same family as (nocturnal) mice and rats but exhibits a strong diurnal niche. As is common in diurnal species, the *R. pumilio* lens acts as a long-pass spectral filter, providing limited transmission of light <400 nm. Conversely, we found strong sequence homologies with the *R. pumilio* SWS and MWS opsins and those of related nocturnal species (mice and rats) whose SWS opsins are maximally sensitive in the near-UV. We continued to assess *in vivo* spectral sensitivity of cone vision using electroretinography and multi-channel recordings from the visual thalamus. These revealed that responses across the human visible range could be adequately described by those of a single pigment (assumed to be MWS opsin) maximally sensitive at ∼500 nm, but that sensitivity in the near-UV required inclusion of a second pigment whose peak sensitivity lay well into the UV range (λ_max_<400 nm, probably ∼360 nm). We therefore conclude that, despite the UV-filtering effects of the lens, *R. pumilio* retains an SWS pigment with a UV-A λ_max_. In effect, this somewhat paradoxical combination of long-pass lens and UV-A λ_max_ results in narrow-band sensitivity for SWS cone pathways in the UV-A range.

## Abstract (precis)

**Summary:** The visual spectral sensitivity of a diurnal rodent, *Rhabdomys pumilio*, is biased against UV-A wavelengths thanks to lens filtering, but not cone spectral sensitivity.

## INTRODUCTION

The vast majority of mammalian retinas contain three classes of photoreceptor: the outer-retinal rods and cones, that mediate form vision in dim and bright conditions, respectively; and the inner-retinal intrinsically photosensitive retinal ganglion cells (ipRGCs), which contribute a lower spatiotemporal resolution representation of the visual environment, supporting aspects of vision as well as an array of non-image-forming light responses (e.g. photoentrainment of the circadian clock). Collectively, these photoreceptors allow organisms to sense and respond to light across the broad variations in illumination that they would commonly encounter in the natural world. Nevertheless, striking variations in mammalian photoreception have emerged throughout evolution, and the spectral sensitivity, anatomical distribution and relative number of each photoreceptor type can vary greatly amongst species (Peichl, 2005). In many cases, this can be attributed to a shift in a species' temporal niche (the time of day at which an animal is most likely to be active), which defines both the quality and quantity of environmental light exposure an animal experiences day to day.

Whether they are diurnal or nocturnal, most mammals have retinas that contain two classes of cone photoreceptor, which are preferentially sensitive to different spectral bands owing to their expression of either short or medium/long wavelength-sensitive photopigments (SWS and MWS, respectively; Jacobs, 1993). Within these classes, there are substantial species differences in spectral sensitivity (Jacobs, 1993; Hunt et al., 2009). There is some evidence to suggest that, at least for SWS pigments, a diurnal temporal niche is associated with a shift in spectral sensitivity towards longer wavelengths (from λ_max_=350–370 nm in nocturnal animals to λ_max_>400 nm in diurnal animals; Emerling et al., 2015). By comparison, the spectral tuning of rod pigments remains largely invariant amongst terrestrial mammals, with λ_max_ values remaining close to 500 nm. As well as the spectral tuning of the visual pigments, pre-receptoral filtering by the lens constrains the spectral sensitivity of mammalian vision (Douglas and Jeffery, 2014), and is strongly associated with temporal niche. Nocturnal species' lenses typically transmit the majority of UV-A light (∼315–400 m), while day-dwelling mammals have a ‘long-pass’ lens which prevents transmission of shorter wavelength light. Indeed, this filtering property can be used as a good predictor of species' temporal niche (Hut et al., 2012). It is thought that limiting UV-A transmission in diurnal species could serve to reduce damage from UV light (van Norren and Gorgels, 2011) and/or to aid higher acuity vision in diurnal species by minimising the impact of chromatic aberration (Lind et al., 2014) and reducing the amount of Rayleigh scatter (Douglas and Jeffery, 2014). The density of cone photoreceptors across the retina also shows dramatic shifts in nocturnal versus diurnal species (Hut et al., 2012), with the latter classically having a greater cone density to match their increased exposure to bright light. Sometimes, that increased density is apparent across the entire retina (such as in the thirteen-lined ground squirrel; Kryger et al., 1998), but it may also occur in spatially localised regions, such as the primate fovea (reviewed by Ahnelt and Kolb, 2000).

Here, we asked whether these general rules hold for a murid rodent – *Rhabdomys pumilio* (the four striped grass mouse) – which is closely related to (nocturnal) mice and rats, but exhibits a strong diurnal niche (Dewsbury and Dawson, 1979; Schumann et al., 2005, 2006). The *R. pumilio* visual system shows several adaptations that are consistent with a diurnal niche, including an increased cone to rod ratio and high cone density (van der Merwe et al., 2018). However, the question of whether the visual system of *R. pumilio* has ‘diurnal’ type spectral sensitivity remains outstanding. Here, we found that, consistent with its diurnal niche, the *R. pumilio* retina is cone rich, and its lens transmits little UV light. In contrast, electrophysiological recordings indicate that *R. pumilio* SWS and MWS cones probably have surprisingly similar spectral sensitivities (predicted λ_max_≈360 nm and 500 nm) to those of their closely related nocturnal counterparts. The outcome is that *R. pumilio* spectral sensitivity is biased against UV-A wavelengths thanks to lens filtering, but not cone spectral sensitivity.

## MATERIALS AND METHODS

### Animals

Animal care was in accordance with the UK Animals, Scientific Procedures Act of 1986, and the study was approved by the University of Manchester ethics committee. Animals were housed on a 12 h:12 h light:dark cycle at 22°C with food and water available *ad libitum*. All experiments were performed in adult *Rhabdomys* *pumilio* (Sparrman 1784) (aged 3–8 months).

### RNA extraction and sequencing

#### Primer design

Primers were designed to amplify the first and last 100–200 bp of SWS and MWS cone opsins from genomic DNA (gDNA). These primers were based on conserved regions of the mouse (*Mus musculus*) and rat (*Rattus norvegicus*) SWS and MWS sequences (genes obtained from NCBI GenBank: 12057 mouse SWS; 14539 mouse MWS; 81644 rat SWS and 89810 rat MWS). Based on gDNA PCR sequencing results, subsequent primers were designed for cloning the full-length coding sequence of *R. pumilio* SWS and MWS cone opsin from retinal cDNA. Each primer included an additional overlapping sequence to allow cloning of the full-length sequence into a linearised plasmid vector using Gibson assembly (Gibson et al., 2009).

#### Genomic DNA PCR

Genomic DNA was obtained from *R. pumilio* ear biopsies. Genomic DNA PCR was performed using Q5 High-Fidelity DNA polymerase (NEB) according to the manufacturer's instructions. The PCR products were run on a 1.5% agarose gel, gel extraction using QIAQuick Gel extraction (Qiagen) was performed on any bands of the expected/appropriate size and fragments were sequenced using Sanger sequencing.

#### Retina RNA extraction and cDNA synthesis

*Rhabdomys pumilio* were killed via cervical dislocation, and both eyes were removed and placed in cold sterile PBS. Each retina was then dissected and placed into a separate sterile RNase- and DNase-free 1.5 ml tube containing 0.5 ml of RNAlater and placed on ice. Retina tissue was stored in RNAlater at −20°C until RNA extraction, which was performed using an RNeasy Mini Kit (Qiagen) according to the manufacturer's instructions, with additional on-column DNase digest (Qiagen) to eliminate potential genomic DNA contamination. Tissue was disrupted using mortar and pestle and homogenised with syringe and needle. The optional extra elution step was also undertaken. RNA was immediately used for cDNA synthesis or stored at −80°C. cDNA synthesis was performed using qScript cDNA Synthesis Kit (QuantaBio) according to the manufacturer's instructions, and stored at −20°C until use.

#### Cloning full-length cone opsin coding sequences

PCR of full-length cone opsin coding sequences was performed on *R. pumilio* retinal cDNA using Q5 High-Fidelity DNA polymerase (NEB), according to the manufacturer's instructions. The following primers were used: for SWS forward 5′-ACTTAAGCTTCACCATGTCGGGAGAGGACGAGT and reverse 5′-TCGAGCGGCCGCTTAGTGAGGGCCAACTTTGCT; and MWS forward 5′-ACTTAAGCTTCACCATGGCCCAAAGGCTTACAGGT and reverse 5′-TCGAGCGGCCGCTTATGCAGGTGACACTGAAG. PCR products were run on a 1.5% agarose gel, and suitable-sized bands were removed and gel extracted using QIAquick Gel Extraction Kit (Qiagen). These were then cloned into a pcDNA3 plasmid vector linearised with HindIII and NotI restriction enzymes using NEB HiFi DNA assembly according to the manufacturer's instructions. A 3 μl sample of the cloning reaction was then transformed in XL10 Gold cells (Agilent) and the plasmid DNA was prepared using a QIAprep Spin miniprep kit (Qiagen). Full-length coding sequence of *R. pumilio* SWS and MWS cone opsin was confirmed by Sanger sequencing of the plasmid insert using the following primers: CMV forward 5′-GGAGGTCTATATAAGCAGAGC and BGH reverse 5′-GGCACCTTCCAGGGTCAAGG.

### Immunohistochemistry

*Rhabdomys pumilio* were anaesthetised with urethane and perfused with 4% paraformaldehyde (methanol free). Eyes were then stored in methanol-free 4% paraformaldehyde prior to further processing. For retinal wholemounts, retinas were dissected from fixed eyes and immunohistochemistry performed on free-floating retinas. For retinal sections, whole eyes were embedded in Historesin and were sectioned at 5 µm thickness. For general histology, fixed eyes were dehydrated through a graded series of alcohols and infiltrated with Technovit 7100 (Historesin TAAB Labs UK). Blocks were sectioned at 5 µm and mounted onto clean slides, stained with Cresyl Violet and coverslipped under DPX. For immunohistochemistry, *R. pumilio* retinas were labelled using polyclonal antibodies against MWS/LWS opsin raised in chicken (PA1-9517, ThermoFisher; 1:250 dilution) and against SWS opsin raised in rabbit (AB5407, Abcam; 1:300 dilution), and left overnight at room temperature. Tissues were then washed and incubated for 2 h in ﬂuorescent secondary antibodies at a dilution of 1:2000 made up of 2% NDS, 3% Triton X-100 and PBS. Sections were imaged with an Axio Imager.D2 upright microscope and captured using a Coolsnap Hq2 camera (Photometrics) through Micromanager software v1.4.23.

### Lens transmission

Lens transmission was assessed as described previously (Douglas and Jeffery, 2014). Briefly, *R. pumilio* were killed (as above) and both eyes removed. Lenses were dissected and immediately frozen (*n*=5 lenses from 3 female *R. pumilio*). After thawing, lenses were rinsed in PBS and mounted in a purpose-built holder in front of an integrating sphere, within a Shimadzu 2101 UVPC spectrophotometer. Transmission at 700 nm was set to 100%, and lenses were scanned at 1 nm intervals from 300 to 700 nm. An equivalent procedure was used to calculate the transmission from *n*=16 mouse eyes.

### Electroretinography

Electroretinograms (ERGs) were recorded from 6 *R. pumilio* (4 female, 2 male), using apparatus and methodology as described previously (Cameron and Lucas, 2009)*.* Anaesthesia was induced with isofluorane (2% in oxygen), and maintained with an intraperitoneal injection of urethane (1.6 g kg^−1^, 30% w/v; Sigma-Aldrich). A topical midriatic (tropicamide 1%; Chauvin Pharmaceuticals, Kingston upon Thames, UK) and hypromellose eye drops were applied to the recording eye prior to placement of a corneal contact lens-type electrode (Sagdullaev et al., 2004). A needle reference electrode was inserted approximately 5 mm from the base of the contralateral eye, and a bite-bar was used for head support and also acted as a ground electrode. Electrodes were connected to a Windows PC via a signal conditioner (model 1902 Mark III, Cambridge Electronic Design, Cambridge, UK) that differentially amplified and filtered (band-pass filter cut-off 0.5–200 Hz) the signal, and a digitiser (model 1401, Cambridge Electronic Design). Core body temperature was maintained at 37°C throughout recordings via a homeothermic heat mat (Harvard Apparatus).

### Visual stimuli: ERG

A CoolLED pe-4000 was used to present 13 stimuli of distinct spectra, with peak wavelengths ranging from 365 to 660 nm. The output was passed through a filter wheel containing a range of neutral density filters, which allowed the light to be modulated across a 6 log unit range. The intensities of each channel were made approximately isoquantal by adjusting the absolute power of each LED, and by using an Arduino Uno to further adjust the pulse width modulation (PWM) of each channel on an 8 bit scale. Stimuli were measured at the corneal plane using a spectroradiometer (SpectroCAL MSII, Cambridge Research Systems, Rochester, UK). All stimuli used were quantified in terms of their photon flux, after accounting for the spectral transmission of the *R. pumilio* lens.

Stimuli were presented following 30 min dark adaptation. Stimuli of different spectra were presented in a pseudorandom order, at 6 intensity levels (moving from dim to bright using a neutral density filter wheel). Dark-adapted stimuli were presented either as a flash (10 ms every 1 s) or as a 32 Hz flicker. Stimuli were also presented at a range of frequencies (1–50 Hz), using a broadband white quartz halogen light (8.6×10^15^ photons cm^−2^ s^−1^) coupled to a mechanical shutter to modulate stimulus frequency. Spectral stimuli were also measured in light-adapted conditions, whereby the filtered output of a quartz halogen light source (500 nm short-pass filter; 4.6×10^14^ photons cm^−2^ s^−1^) was superimposed upon narrowband spectra. Stimuli were combined using a bifurcated mixed fibre optic with opal diffuser at the output.

A further set of stimuli were designed using the principles of receptor silent substitution (as used previously; Allen et al., 2014; Allen and Lucas, 2016). Briefly, a pair of spectra was generated using a combinations of CoolLED channels, which were designed to be isoluminant for *R. pumilio* MWS and SWS opsins (using putative λ_max_ of 360 and 500 nm; stimuli nominally termed ‘stimulus’ and ‘background’ spectra, respectively). A second pair of spectra was generated, designed to be isoluminant for *R. pumilio* MWS opsin but present 99% Michelson contrast for the putative *R. pumilio* SWS opsin. ERGs were then recorded whilst transitions between these pairs of spectra were presented to the *R. pumilio* eye (10 ms flash of stimulus spectrum interleaved with 990 ms of background spectrum).

For flash ERGs, b-wave amplitudes were measured relative to the baseline (value at flash onset) or, when measurable, relative to the trough of the preceding a-wave. For all flicker ERGs, a mean of each 1 s cycle was taken; the average amplitude of the first 4 peak to trough responses was measured, with a period equal the stimulus frequency.

### *In vivo* electrophysiological recordings in the dorsal lateral geniculate nucleus (dLGN)

*In vivo* electrophysiological recordings were performed in 3 *R. pumilio*, using methods described previously (Brown et al., 2012)*.* Anaesthesia was induced with 2% isofluorane in oxygen, and maintained with an intraperitoneal injection of urethane (1.6 g kg^−1^, 30% w/v; Sigma-Aldrich). A topical mydriatic (as with ERGs) and mineral oil (Sigma-Aldrich) were applied to the left eye prior to recording. After placement into a stereotaxic frame, the *R. pumilio* skull was exposed and a small hole drilled ∼2.5 mm posterior and ∼2.5 mm lateral to bregma. A 256-channel recording probe (A4x64-Poly2-5mm-23s-250-177-S256, NeuroNexus Technologies, Inc., Ann Arbor, MI, USA) consisting of 4 shanks spaced 200 µm apart, each with 64 recording sites, was lowered a depth of ∼3–3.5 mm into the brain, targeting the *R. pumilio* dLGN. Broadband neural signals were then acquired using a SmartBox recording system (NeuroNexus Technologies, Inc.), sampling at 20 kHz. Following recordings, data from each of the four electrode shanks were pre-processed by common median referencing, high-pass filtered at 250 Hz and then passed to an automated template-matching-based algorithm for single unit isolation (Kilosort; Pachitariu et al., 2016preprint). Isolated units were then extracted as virtual tetrode waveforms for validation in Offline Sorter (V3, Plexon, Dallas, TX, USA). Here, unit isolation was confirmed by reference to MANOVA *F* statistics, J3 and Davies-Bouldin validity metrics and the presence of a distinct refractory period (greater than 1.5 ms) in the interspike interval distribution.

Spike sorted data were further analysed in MATLAB R2018a (The MathWorks). Peri-event response histograms for each stimulus were calculated (250 ms bins; mean of 20 trials) with response quantified as the maximum absolute change in spike rate during or in the 1 s following visual stimulation relative the baseline (mean spike rate in the 1 s prior to visual stimulation). This approach therefore captured both ON and OFF responses. To identify significant changes in spike rate, responses calculated in this manner were compared with the distribution of ‘response’ values determined from 1000 repeats using trial×trial time-shuffled spike counts. The mean of the shuffled response distribution was subsequently subtracted from the actual response such that, on average, a lack of response would give a value of 0 spikes s^−1^.

### Visual stimuli: dLGN

Experiments employed a custom-built light source (all components from Thorlabs, Ely, UK) consisting of a cold white LED (MCWHLP1) with an automated narrowband filter wheel (FW102C, loaded with bandpass filters at 425, 450, 495, 530, 560 and 600 nm; bandwidth: ±10 nm) and a second adapting cold white LED fitted with selectable blue and yellow broadband filters (centred at 450 and 550 nm, respectively; bandwidth: ±40 nm). LED intensity was controlled by current modulation via T-Cube drivers and, where required, neutral density filters. Output from the adapting and probe sources was then combined and delivered to the subject via a randomised bifurcated light guide (E436, Dolan Jenner, Boxborough, MA, USA) whose output ferrule (6.3 mm diameter) was positioned 5 mm from the contralateral eye and enclosed by an internally reflective plastic cone to provide approximately full-field illumination. Stimulus measurements were performed using a calibrated spectroradiometer as above.

Stimulus delivery was controlled using LabVIEW (National Instruments, Austin, TX, USA). Animals were first dark adapted for 30 min. To assess rod spectral sensitivity we delivered dim 1 s narrowband flashes across the 6 available test wavelengths at 6 different intensities (∼10^8^ to 10^10.5^ photons cm^−2^ s^−1^; 20 repeats per intensity/wavelength). The order of stimulus delivery was randomised according to wavelength between each of the 20 trials but scheduled such that the lowest intensity trials were completed first and the highest intensity trials last. Subsequent assessment of cone spectral sensitivity was performed similarly except in this case we employed 7 rather than 6 intensities at each wavelength (∼10^12^ to 10^14.5^ photons cm^−2^ s^−1^) and stimuli were superimposed on a rod-saturating short followed by long wavelength background (∼10^13.5^ rod effective photons cm^−2^ s^−1^ for both).

Note that a different set of spectral stimuli were used with dorsal lateral geniculate nucleus (dLGN) and ERG experiments, owing to technical constraints. Nevertheless, our experiments were carefully designed with this constraint in mind. Thus, while our ERG studies provide a comprehensive description of sensitivity across a wide wavelength range, our dLGN experiments were designed to determine whether: (1) *R. pumilio* use a dedicated short wavelength-sensitive receptor (revealed by the presence of neurones responsive to short but not longer wavelength stimuli); and (2) the SWS cone has λ_max_ in the UV range, by determining whether responses to shorter wavelength stimuli survive application of a background that should suppress responses from any pigment with peak sensitivity >400 nm.

### Analysis of spectral stimuli

All spectral stimuli used were quantified in terms of their absolute photon flux, as described previously (Allen et al., 2014). Two approaches were used to predict the spectral sensitivity of *R. pumilio* cone opsins from ERG studies. First, irradiance–response functions were plotted for each wavelength, and data fitted with a sigmoidal dose–response curve: *y*=*a*+(*b*−*a*)0/(1+10^{({\rm logEC}_{50−x})}), where *a* is the base and *b* is the top of the curve. For wavelengths that evoked responses at only one or two intensities, data were not included because of ambiguous curve fits. Curve parameters were fixed across each stimulus, such that the only free parameter was the EC_50_. EC_50_ values were then plotted as a function of wavelength. Note, however, that bandwidth was often >25 nm (FWHM), and hence when plotting response amplitude as a function of wavelength, the peak wavelength of each channel was used. The best-fitting λ_max_ was calculated using a Govardovskii nomogram (Govardovskii et al., 2000).

In a second approach, we modelled the sensitivity of two hypothetical cone opsins to find the combination of λ_max_ values that best described physiological responses. First, we calculated the predicted photon fluxes of SWS and MWS opsins with a range of hypothetical λ_max_ values (ranging from 360 to 420 nm and 470 to 530 nm, respectively), and with a range of weighted contributions, using the following formula:(1)
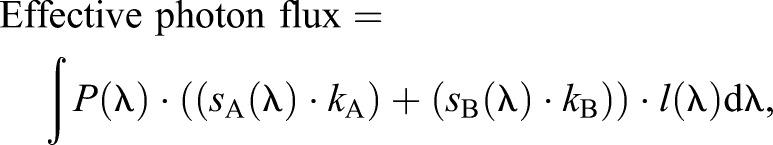
where *P*(λ) is spectral irradiance in photons cm^−2^ s^−1^ nm^−1^, *s*_A_(λ) and *s*_B_(λ) are pigment spectral sensitivity approximated by the Govardovskii visual template (for two pigments, A and B, incorporating a beta peak; Govardovskii et al., 2000), *k*_A_ and *k*_B_ are relative weighting factor (for pigments A and B), and *l*(λ) is *R. pumilio* lens transmission.

For each combination, we plotted the response at each intensity at each wavelength as a function of effective rate of photon flux for a weighted combination of the two hypothetical pigments. We then tested which combination of λ_max_ and weighted contribution to the evoked response allowed the combined intensity–response curve to be best fitted by a single curve.

For dLGN recordings, we used a qualitatively equivalent (but computationally faster) approach to estimate single cell spectral sensitivity. Hence, here spectral sensitivity was determined by calculating irradiance–response relationships across all test wavelengths according to the effective photon flux experienced by a single opsin with arbitrary λ_max_ in the range 350–600 nm (taking into account *R. pumilio* lens transmission). We then calculated the four-parameter sigmoid curve that best fitted that irradiance–response relationship and determined the percentage variance in the data accounted for by that fit. Analysis of a subset of cells using the first approach described above for ERG produced identical λ_max_ estimates.

To add context to the transmission characteristics of the *R. pumilio* lens, we calculated the impact of the lens transmission on the effective photon flux of *R. pumilio* and mouse SWS and MWS pigments in natural daylight [using spectra measured in Manchester, UK (53°21′N, 2°16′W, elevation of 78 m), 2 weeks after the summer solstice, at a solar angle of +30 deg published previously (Allen et al., 2014)].

## RESULTS

### *Rhabdomys pumilio* SWS and MWS cone opsins

**Fig. 1.**
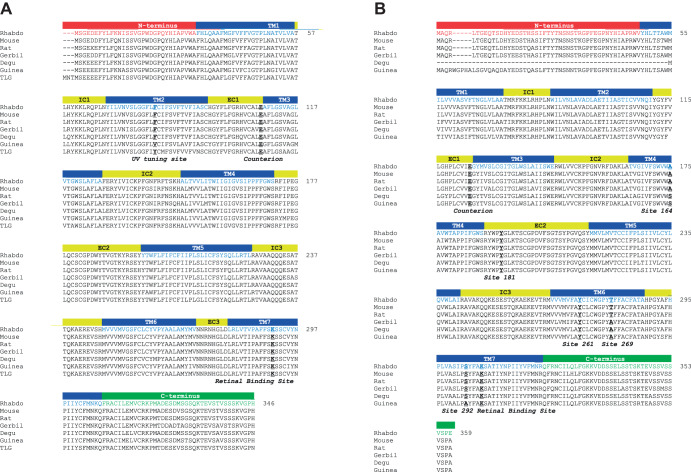
**Alignment of *Rhabdomyspumilio* SWS and MWS opsins with sequences of rodent species.** (A) SWS opsin of *R.* *pumilio* (Rhabdo) and of the following species: *Mus musculus* (Mouse, NP_031564.1), *Rattus norvegicus* (Rat, NP_112277.1), *Meriones unguiculatus* (Gerbil; XP_021517546.1), *Octodon degus* (Degu; XP_004642783.1), *Cavia porcellus* (Guinea pig; NP_001166229) and *Ictidomys tridecemlineatus* [thirteen-lined ground squirrel (TLG); XP_021578083.1]. *Rhabdomys* *pumilio* SWS opsin structure is based on mouse SWS1 opsin structure and is shown by labelled coloured bars: TM, transmembrane domain; IC, intracellular loops; EC, extracellular loops. Key sites are shown in bold and underlined: UV tuning site 86, counter-ion site 113, and retinal binding site 296. (B) MWS opsin of *R.* *pumilio* and of the following species: *M. musculus* (NP_032132.1), *R. norvegicus* (NP_446000.1), *M. unguiculatus* (XP_021484930.1), *O. degus* (XP_023561139.1), *C. porcellus* (NP_001166460.1) and *I. tridecemlineatus* (AAW29517.1). *Rhabdomys* *pumilio* MWS opsin structure is based on mouse MWS opsin structure and is shown by labelled coloured bars as in A. Key sites are shown in bold and underlined: counter-ion site 113; retinal binding site 296; and LWS/MWS spectral tuning sites: 164, 181, 261, 269 and 292. Numbering of key sites is based on bovine rod opsin. All alignments were performed using MAFFT (Katoh and Standley, 2013).

We were able to amplify full-length sequences for both SWS and MWS cone opsins from *R. pumilio* retinal cDNA (Fig. 1). The predicted sequences for these two opsins are 346 and 359 amino acids long, respectively. When aligned against the corresponding opsin sequence from multiple other rodent species [*Mus musculus* (mouse), *Rattus norvegicus* (rat), *Octodon degus* (degu), *Meriones ungiuculatus* (gerbil), *Cavia porcellus* (guinea pig) and *Ictidomys tridecemlineatus* (thirteen-lined ground squirrel)], *R. pumilio* opsins showed highest sequence homology with mouse and rat (∼96% and 97%, respectively) as predicted for the phylogeny of these species (Blanga-Kanfi et al., 2009) (sequence homologies are summarised in Table 1).

**Table 1.**
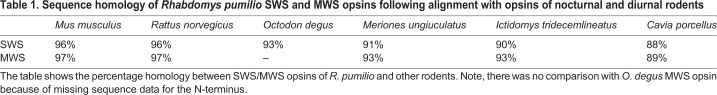
**Sequence homology of *Rhabdomys pumilio* SWS and MWS opsins following alignment with opsins of nocturnal and diurnal rodents**

A high degree of homology was retained at known spectral tuning sites between cone opsins of *R. pumilio* and other rodent species. The *R. pumilio* cone opsin sequences had highly conserved opsin characteristics, such as the chromophore binding site, Lys296, and retinal counter-ion, Glu113 (numbering throughout based on bovine rod opsin). When we examined known spectral tuning sites, we found the *R. pumilio* sequences were most similar to those of rats and mice. For example, *R. pumilio*, mice and rats all possessed phenylalanine at position 86, which has been established as an important site for UV spectral tuning in vertebrates (Cowing et al., 2002; Fasick et al., 2002; Hunt et al., 2004, 2007). When we examined MWS opsin sequences at the ‘five sites’ involved in spectral tuning differences between MWS/LWS photopigments (Yokoyama and Radlwimmer, 1998), we found these residues in *R. pumilio* cone opsin were identical to rat and mouse sequences (Ala164, Tyr181, Glu261, Tyr269 and Ser292, respectively). Initial analysis of the predicted protein sequence suggests spectral tuning of *R. pumilio* cone opsins may be similar to that of rat or mouse.

**Fig. 2.**
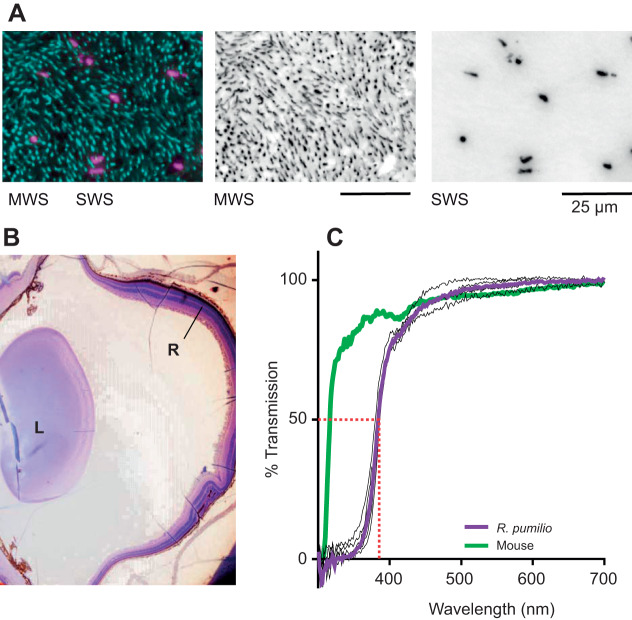
**Anatomical features of *R.pumilio* retina and transmission of *R.pumilio* lens.** (A) Immunohistochemistry for MWS (cyan) and SWS (pink) opsins on a retinal wholemount. Left: overlay; middle: MWS opsin; right: SWS opsin. Scale bars: 25 µm. (B) Sagittal section of *R.* *pumilio* eye following Cresyl Violet staining, showing lens (L) and neural retina (R). (C) Spectral transmission of 5 *R.* *pumilio* lenses (black lines) and group mean (purple line), and the group mean of 16 mouse lenses (green line), from 300 to 700 nm. All values were normalised to transmission at 700 nm.

Immunohistochemical labelling of cone opsins in the *R. pumilio* retina identified both SWS- and MWS-expressing cones (Fig. 2A). Unlike in mice (Applebury et al., 2000), we saw no evidence of cones co-expressing the two types of opsin. A recent analysis of cone density in the *R. pumilio* retina (van der Merwe et al., 2018) reported that MWS-expressing cones were more numerous than SWS-expressing cones across the retina, and that both cones showed lower density in the periphery. We did not undertake an extensive validation of those observations, but our immunocytochemistry results did confirm that MWS cones were substantially more numerous that SWS cones (Fig. 2A).

### Spectral transmission of *R. pumilio* lens

As a prelude to descriptions of *R. pumilio* spectral sensitivity *in vivo*, we measured spectral transmission of the *R. pumilio* lens across the UV-visible wavelength range (5 adult *R. pumilio* lenses; mean diameter along the optic axis 2.6 mm; Fig. 2B). We found that the *R. pumilio* lens acts as a long-pass filter, allowing efficient transmission of wavelengths ≥400 nm (50% transmission at 383.5±1.2 nm; Fig. 2C). The *R. pumilio* lens is therefore substantially less transmissive for UV light than mouse or rat lenses, both of which have a 50% transmission point at a wavelength approximately 70 nm shorter (Douglas and Jeffery, 2014).

### *Rhabdomys pumilio in vivo* spectral sensitivity: ERG

**Fig. 3.**
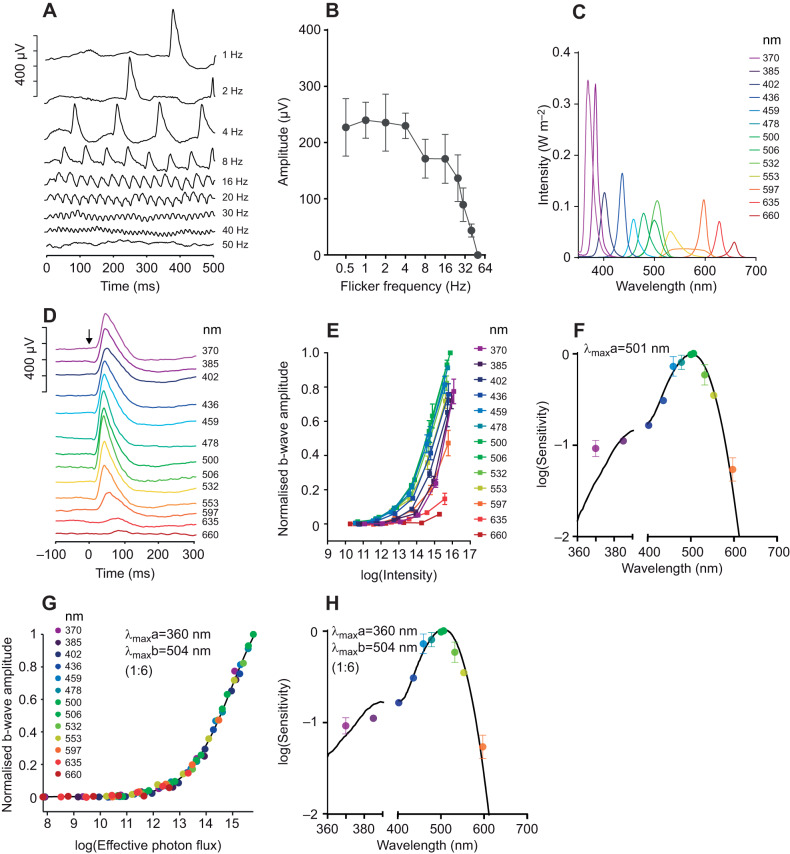
**Spectral sensitivity of** **electroretinogram (ERG) responses to a dark-adapted flash.** (A) Representative ERG responses to flicker stimuli of different frequencies**.** (B) Response amplitude of flicker stimuli of different frequencies (*n*=3; means±s.e.m.). (C) Spectral power distribution of 13 stimuli used to track spectral sensitivity. (D) Representative flash ERGs for spectral stimuli presented at maximum intensity. (E) Normalised b-wave amplitude for spectral stimuli presented at up to 6 intensities (photons cm^−2^ s^−1^; *n*=3; means±s.e.m.). (F) Mean±s.e.m. EC_50_ values plotted as a function of wavelength (central peak of each channel). Black line shows the best-fitting spectral sensitivity function (accounting for lens transmission) to describe these data (λ_max_=501 nm; *R*^2^=0.978). Note that 635 and 660 nm data points were excluded given the low sensitivity to these wavelengths. (G) Response amplitude as a function of effective photon flux (photons cm^−2^ s^−1^) for the best-fitting nomogram/pair of nomograms. In this case, the best fit was composed of two pigments with λ_max_ of 360 and 504 nm, at a ratio of 1:6. The curve fit has an *R*^2^ of 0.999. (H) Spectral sensitivity function of the best-fitting nomogram/pair of nomograms (used to generate the *x*-axis in G). EC_50_ values were replotted from F for comparison.

We continued to assess the spectral sensitivity of *R. pumilio* vision using the ERG. To begin, we established the basic temporal response characteristics of the *R. pumilio* ERG by recording dark-adapted responses to full-field flicker across a range of frequencies (ranging from 1 to 50 Hz; 100% contrast). In such conditions, ERGs were measurable at frequencies ≤40 Hz (Fig. 3A,B). Mice and rats are able to track flicker up to a similar frequency when measured in cone-isolating (light-adapted) conditions (Krishna et al., 2002; Qian et al., 2008). Across this frequency range, under these conditions, one would expect a switch from rod+cone towards predominantly cone-based responses. We therefore recorded ERG spectral sensitivity at low and high temporal frequencies (1 and 32 Hz) by recording responses to 13 spectrally distinct (∼isoquantal) stimuli across a 6-log unit range of light intensity (Fig. 3C). When presented at 1 Hz, we were able to record measurable ERGs to the brightest flash across all wavelengths (Fig. 3D). Irradiance–response curves revealed maximum sensitivity around 500 nm (Fig. 3E), which would be typical for a rod-driven response across other mammalian species. However, when we quantified relative sensitivity across wavelengths, we found higher sensitivity to the shortest wavelength tested than predicted for a single photopigment with λ_max_ around 500 nm when accounting for pre-receptoral filtering by the *R. pumilio* lens (Fig. 3F). The simplest explanation is that there is some intrusion of SWS cones to this response. We therefore asked whether inclusion of an additional short wavelength pigment improved the fit of the data. Given that intensity–response functions had a qualitatively similar form across the wavelength range (Fig. 3E), we applied a simple modelling process in which we asked what combination of pigments would be required to predict the pattern of relative sensitivity across wavelengths. In brief, we attempted to describe measured sensitivity across all wavelengths by calculating the effective photon flux for a system in which responses were elicited by the combined activity of two opsin pigments with different spectral sensitivity. To achieve the objective of having the same response to a given effective photon flux irrespective of wavelength, we varied two parameters: the peak sensitivity (λ_max_) of each opsin, and their relative contribution to the evoked response for a theoretical spectrally neutral light source (contribution weighting ratio). This method achieved a good fit for the data using pigments with predicted λ_max_ of 360 and 504 nm at 1:6 contribution weighting (Fig. 3G). The predicted spectral sensitivity function for this combination of opsins, accounting for the filtering effects of the lens, is shown in Fig. 3H.

**Fig. 4.**
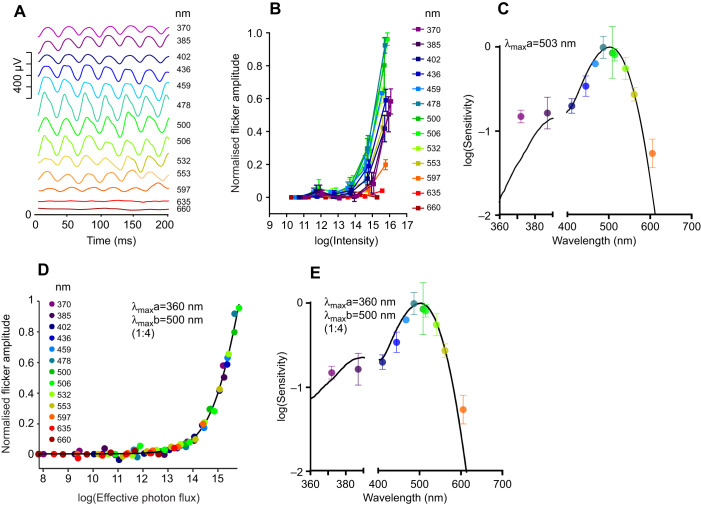
**Spectral sensitivity of ERG responses to a 32 Hz flicker.** (A) Representative ERG responses to maximum intensity spectral stimuli presented as a 32 Hz flicker. (B) Normalised response amplitude for spectral stimuli presented at up to 6 intensities (photons cm^−2^ s^−1^; *n*=3; means±s.e.m.). Note that 635 and 660 nm data points were excluded given the low sensitivity to these wavelengths. (C) Mean±s.e.m. EC_50_ values plotted as a function of wavelength (central peak of each channel). Black line shows the best-fitting spectral sensitivity function (accounting for lens transmission) to describe these data (λ_max_=503 nm; *R*^2^=0.969). (D) Response amplitude as a function of effective photon flux (photons cm^−2^ s^−1^) for the best-fitting nomogram/pair of nomograms. In this case, the best fit was composed of two pigments with λ_max_ of 360 and 500 nm, at a ratio of 1:4. The curve fit has an *R*^2^ of 0.996 (*n*=3). (E) Spectral sensitivity function of the best-fitting nomogram/pair of nomograms (used to generate the *x*-axis in D). EC_50_ values replotted from C for comparison.

The appearance of a short wavelength cone component to the 1 Hz response might have been expected given the cone-rich nature of the *R. pumilio* retina. More surprising was that there was no requirement to account for an additional MWS cone contribution to the response. The most likely explanation is that the MWS opsin has maximal sensitivity around 500 nm, near peak sensitivity of the 1 Hz response. Extending our spectral sensitivity investigation to conditions favouring cones (32 Hz flicker) confirmed that this is indeed the case (Fig. 4A,B). Once again, responses could be recorded across the wavelength range and peak sensitivity lay around 500 nm (Fig. 4C). Applying the same criteria used for 1 Hz responses revealed that the data could be adequately fitted by a 1:4 combination of opsins with λ_max_ of 360 and 500 nm (Fig. 4D,E). The prediction that MWS opsin has a greater impact on flicker spectral sensitivity than SWS opsin is consistent with our own (Fig. 2) and published (van der Merwe et al., 2018) observations that MWS cones are more numerous in the *R. pumilio* retina. The reduction in this ratio compared with that recorded at 1 Hz (at which it is 1:6; Fig. 3G) probably reflects the additional contribution of rods to middle/long wavelength sensitivity at low temporal frequencies.

**Fig. 5.**
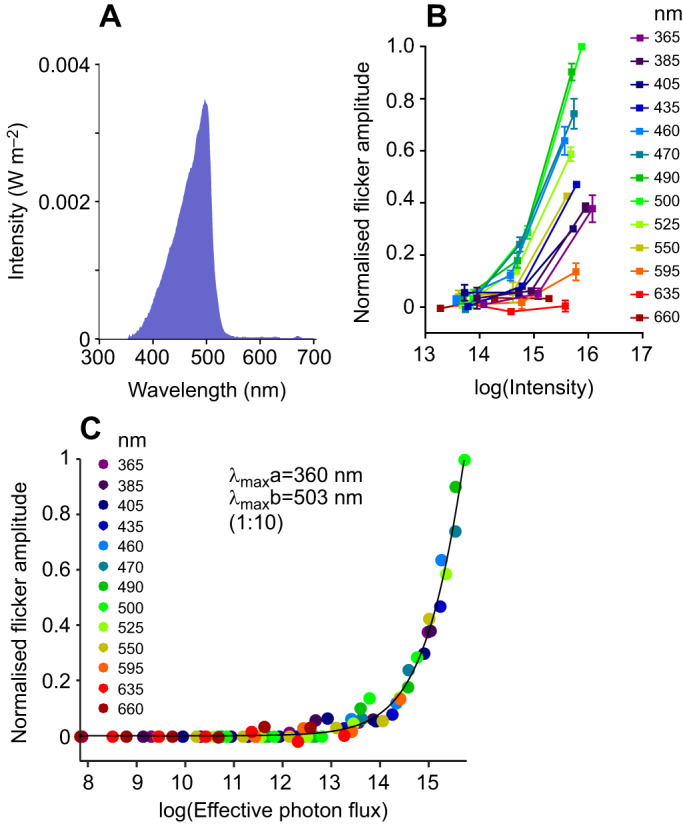
**Spectral sensitivity of ERG responses in light-adapted conditions.** (A) Spectral power distribution of background light. (B) Normalised response amplitude for 32 Hz flicker, measured across 3 intensities (photons cm^−2^ s^−1^) for each spectral stimulus, and in the presence of an adapting background light (*n*=3; means±s.e.m.). (C) In the presence of background light, response amplitude as a function of effective photon flux (photons cm^−2^ s^−1^) was best fitted with a pair of nomograms with λ_max_ of 360 and 503 nm, at a ratio of 1:10 (*R*^2^=0.992; *n*=3).

As a further confirmation that the high-frequency flicker stimuli faithfully reported cone visual sensitivity, we repeated those recordings under light-adapted conditions with the goal of saturating any residual rod-evoked responses. We recorded flicker ERG responses following adaptation to (and in the presence of) a broad-spectrum background light covering the spectral sensitivity range expected for rods and the two putative cone pigments (Fig. 5). The background light had no discernible impact on flicker ERG spectral sensitivity. Thus, modelling the data as the output of a combination of two pigments (as above) returned λ_max_ of 360 and 503 nm. Note that the adapting light is predicted to differentially impact middle/long wavelength pigments, which probably explains the increase in contribution weighting of SWS versus MWS opsins to 1:10 for this high-frequency flicker stimulus compared with that recorded under dark-adapted conditions.

**Fig. 6.**
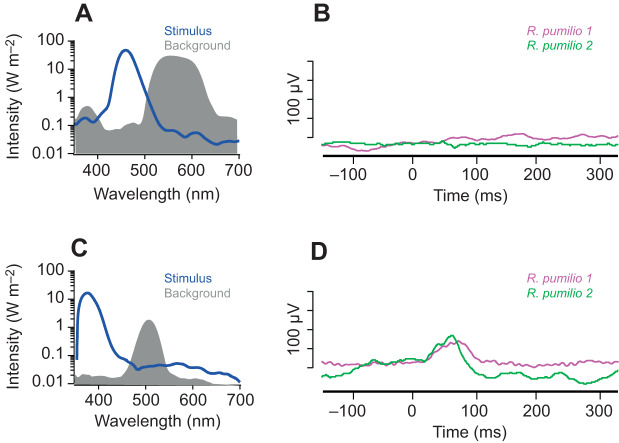
**Silent substitution ERG responses.** (A) Pair of spectra (stimulus and background) designed to be isoluminant for putative *R.* *pumilio* SWS and MWS cone opsins (accounting for lens transmission). (B) ERG response of two *R.* *pumilio* to a transition between the spectral pair (10 ms flash of stimulus spectrum, interleaved with 990 ms of background spectrum). (C) Pair of spectra designed to be isoluminant for putative *R.* *pumilio* MWS cone opsin, but presenting 99% contrast for putative *R.* *pumilio* SWS cone opsin (accounting for lens transmission). (D) ERG response of two *R.* *pumilio* to a transition between this spectral pair (10 ms flash of stimulus spectrum, interleaved with 990 ms of background spectrum).

As lens filtering of UV light makes it difficult to unambiguously record a short wavelength peak in the composite ERG attributable to SWS cones, we set out to use the approach of receptor silent substitution to record an isolated SWS response. First, we generated a pair of spectrally distinct stimuli that were designed to be isoluminant for these two proposed photopigments (<5% Michelson contrast). In accordance with our prediction, transitions between these two spectra (10 ms/1000 ms) drove no measurable ERG response (Fig. 6A,B). We then designed a pair of stimuli that were isoluminant for putative MWS cones, but presented contrast for an SWS pigment. Transitions between these spectra elicited a flash ERG response (Fig. 6C,D). This confirms that the *R. pumilio* ERG cannot be accounted for by a single pigment with λ_max_ around 500 nm.

### *Rhabdomys pumilio in vivo* spectral sensitivity: dLGN

Our ERG data describe the spectral sensitivity of *R. pumilio* cone visual responses, but the lens filtering of UV light makes the contribution of SWS opsin to the composite spectral sensitivity profile slight. As additional confirmation that there was indeed an independent short wavelength-sensitive contribution to visual responses, we turned to recordings in the dLGN, in which a subset of individual units might reasonably be expected to convey information only from SWS cones. To this end, we performed large-scale multielectrode recordings from the *R. pumilio* dLGN (256-channel recordings from 3 *R. pumilio*). Using this approach, we were able to describe the spectral sensitivity of individual neurons, as opposed to a composite response, and therefore test the prediction that some neurons would exhibit specific short wavelength sensitivity in the UV range.

To determine the functional spectral sensitivity of the isolated neurons, we presented a series of 6 spectrally distinct stimuli (randomised order) as 1 s full-field flashes across a ∼2 log unit range of intensity (flash intensities ∼10^12^ to 10^14.5^ photons cm^−2^ s^−1^; note that a different set of spectral stimuli were used with dLGN and ERG experiments, owing to technical constraints). Light flashes were superimposed on a short or long wavelength background light (450 and 550 nm, respectively; bandwidth ±40 nm), designed to suppress rod activity (∼10^13.5^ effective photons cm^−2^ s^−1^), and bias responses in favour of cones. The backgrounds also represented an opportunity to confirm the UV peak of SWS opsin sensitivity. Thus, while the two backgrounds had equivalent effective intensity for MWS cones (respectively 10^13.4^ and 10^13.6^ effective photons cm^−2^ s^−1^), even the shorter wavelength should have negligible impact on SWS cones with a λ_max_ of 360 nm (10^10.5^ effective photons cm^−2^ s^−1^). In this way, we would expect both wavelengths to suppress MWS more than SWS cone responses. Conversely, if the *R. pumilio* SWS opsin had λ_max_ >400 nm, we would expect marked suppression of short wavelength responses in the presence of our short (but not long) wavelength background light.

**Fig. 7.**
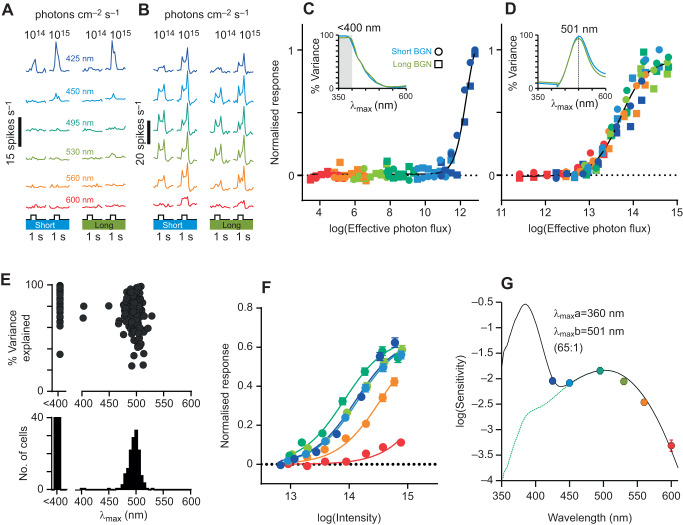
**Spectral sensitivity of dorsal lateral geniculate nucleus (dLGN) neuron responses under light adaptation.** (A,B) Representative responses of two *R.* *pumilio* dLGN neurons to 1 s moderate and bright light flashes (means of 20 trials) of varying wavelength under short and long wavelength adaptation (450 and 550 nm, respectively, bandwidth ±40 nm; 10^13.5^ rod-effective photons cm^−2^ s^−1^). (C,D) Irradiance–response relationships for neurons in A and B with irradiance quantified according to the effective (lens-corrected) photon flux (photons cm^−2^ s^−1^) for a single opsin with a λ_max_ of 360 nm (C) and 501 nm (D). Note the differing range on the *x*-axes. Insets show spectral sensitivity estimates under short and long wavelength adaptation; note that tested wavelengths offered little discriminatory power for λ_max_ <400 nm. (E) Population spectral sensitivity estimates (*n*=189 neurons, based on average responses across short and long wavelength backgrounds) showing best-fitting single opsin λ_max_ and corresponding response variance explained. (F) Normalised mean±s.e.m. irradiance–response relationships for all dLGN cells that responded to intensity (photons cm^−2^ s^−1^) under light-adapted conditions, fitted with 4-parameter sigmoid curves. (G) Sensitivity estimates (from F), best fitted to a pair of *R.* *pumilio* lens-corrected opsin templates with λ_max_ of 360 and 501 nm at a ratio of 65:1 (*R*^2^=0.99).

With this approach, we were able to describe the spectral sensitivity of light-evoked responses from 189 single neurons. Two distinct patterns of spectral sensitivity emerged: one group of neurons (*n*=42) exhibited strong responses to only the shortest wavelength (425 nm) flashes (Fig. 7A,C), consistent with an SWS cone-dependent origin. The other group of neurons (*n*=147) responded to all wavelengths with a sensitivity consistent with a strong MWS cone bias (Fig. 7B,D). Given that, for both groups of neurons, responses were essentially identical under short and long wavelength backgrounds (Fig. 7A–D), subsequent analyses used the average response across the two backgrounds. Corresponding single opsin spectral sensitivity estimates for individual cells (Fig. 7E) were strongly clustered around ∼500 nm (mean±s.e.m. 501±3 nm for the 5 best-fitting cells; >95% variance explained) or <400 nm (tested wavelengths did not provide reliable discrimination at shorter wavelengths but results are fully compatible with λ_max_=360 nm). Consistent then with the ERG data described above, across the entire population of dLGN neurons, overall spectral sensitivity could be very well explained by a combination of two pigments with λ_max_ of 360 and 501 nm (Fig. 7F,G). In this case, however, effective SWS cone contributions were much stronger than observed in ERG studies (Fig. 7G, best fit ratio of 65:1; *R*^2^=0.99). This can be explained by the presence of the adapting background lights, which are predicted to suppress sensitivity of pigments with λ_max_ >400 nm (in this case MWS but not UVS cone opsins). This observation (and the similarity in responses under the two tested backgrounds) thus represents further support for the hypothesis of a UV peak sensitivity for *R. pumilio* SWS opsin.

**Fig. 8.**
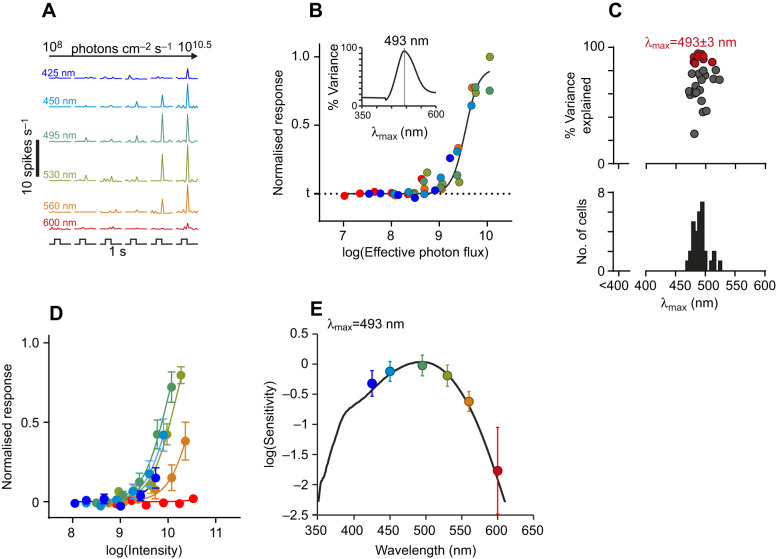
**Spectral sensitivity of dLGN neuron responses under scotopic conditions.** (A) Representative responses of a *R.* *pumilio* dLGN neuron to 1 s dim light flashes (means of 20 trials) of varying intensity and wavelength under dark-adapted conditions. (B) Irradiance–response relationship for neuron in A with irradiance quantified according to the effective photon flux (photons cm^−2^ s^−1^) for a single opsin with a λ_max_ of 493 nm (corrected for *R.* *pumilio* lens transmission). Inset shows spectral sensitivity estimate (quantified as the percentage variance in effective irradiance–response curves explained by the best-fitting 4-parameter sigmoid curve). (C) Population spectral sensitivity estimates showing the best-fitting single opsin λ_max_ and corresponding response variance explained (as in B). (D) Normalised mean±s.e.m. irradiance–response relationships for cells whose responses to light intensity (photons cm^−2^ s^−1^) were best explained by a single opsin (>80% variance explained, highlighted in red in C), fitted with 4-parameter sigmoid curves. (E) Sensitivity estimates (from D), fitted to a *R.* *pumilio* lens-corrected opsin template with λ_max_ of 493 nm (*R*^2^=0.99).

We also applied this protocol under scotopic conditions following 30 min dark adaptation, and using stimuli 4 log units dimmer than in light-adapted conditions (flash intensities ∼10^8^ to 10^10.5^ photons cm^−2^ s^−1^). In these conditions, we would expect rod photoreception to dictate the sensitivity of the resulting responses. Across 31 neurons that exhibited robust responses under these conditions (Fig. 8A), we determined which single opsin could best account for the observed pattern of responses, using an approach equivalent to that described above (Fig. 8B). As expected from the rod spectral sensitivity of other mammals, the estimates of λ_max_ were tightly clustered at values just below 500 nm (Fig. 8C). Among these cells, the group whose responses could be best explained by the spectral sensitivity of a single opsin (>80% variance in responses accounted for) had a λ_max_ of 493±3 nm (mean±s.e.m., *n*=7; Fig. 8C–E).

### Estimating the impact of the *R. pumilio* lens on SWS and MWS opsins

Our data all support the notion that the lens of *R. pumilio* has a low transmission for wavelengths <400 nm, but an SWS opsin with λ_max_ of ∼360 nm. To provide some real-world context to these values, we assessed the expected excitation of *R. pumilio* and mouse SWS and MWS opsins in daylight. Using an environmentally measured spectrum of sunlight, we asked how the predicted excitation of SWS and MWS opsins was impacted by lens transmission in *R. pumilio* and mice (Fig. 2, Table 2). We observed a reduction of approximately 70% in the excitation of the *R. pumilio* SWS opsin, compared with ∼15% in the mouse (for MWS opsins in each species, the impact of lens transmission is minimal). Note, however, that despite the increased impact of the *R. pumilio* lens on the SWS opsin, its relative excitation in daylight remained well above threshold (and within the range of Weber adaptation), suggesting reasonable UV sensitivity despite low transmission of the *R. pumilio* lens for wavelengths <400 nm.

**Table 2.**
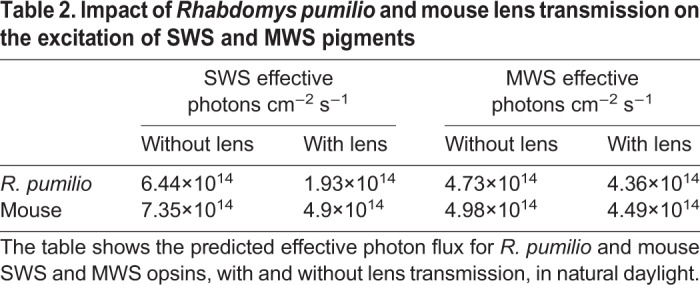
**Impact of *Rhabdomys pumilio* and mouse lens transmission on the excitation of SWS and MWS pigments**

## DISCUSSION

We set out to examine the *in vivo* spectral sensitivity of the visual system of the diurnal rodent *R. pumilio* and establish the extent to which its visual system is consistent with its diurnal lifestyle. The *R. pumilio* retina contains two classes of cones, rods and ipRGCs. As with other diurnal species, the *R. pumilio* eye contains a cone-rich retina. Both amino acid sequence analysis and electrophysiological recordings are consistent with the conclusion that the spectral sensitivity of SWS and MWS cones is similar to that of closely related nocturnal species. However, the presence of a long-pass lens appears to greatly impact sensitivity to shorter wavelengths, producing anomalous narrow-band spectral tuning of *R. pumilio* SWS cones.

Our functional data are consistent with previous reports that the *R. pumilio* retina is cone dominated (>50% cone photoreceptors; van der Merwe et al., 2018). We reveal robust cone-evoked ERG responses that can track stimuli inverting at high temporal frequencies (≤40 Hz), and robust, large-amplitude responses across the high light levels tested here. Similarly, dLGN responses are more numerous and robust under cone-favouring photopic conditions. Previous immunohistochemical analyses have established that the *R. pumilio* retina contains two cone opsins (SWS and MWS; van der Merwe et al., 2018), with higher expression of MWS opsin (as often observed in such rodent species). We were able to clone and sequence both *R. pumilio* cone opsins, and a comparison with related rodent species (both nocturnal and diurnal) revealed very close sequence homology with mouse and rat SWS/MWS opsins, predicting similarities in their spectral sensitivity. In agreement with this prediction, both ERG and dLGN recordings revealed that, while sensitivity across the human visible spectrum was consistent with that of a single photopigment with λ_max_ around 500 nm, responses to near-UV wavelengths were anomalously sensitive. One obvious potential origin for high UV sensitivity is intra-ocular fluorescence. However, this does not provide an adequate explanation for our data, as we found that a subset of dLGN neurons respond only to 425 nm, not the longer wavelengths that should be the product of fluorescence. Moreover, a background light that should suppress responses from any SWS pigment with peak sensitivity in the human visible range did not impact responses to the shorter wavelengths. These findings argue that there is indeed a photoreceptor specifically sensitive to very short wavelength light and that the *R. pumilio* SWS pigment has a λ_max_ of <∼400 nm.

Direct electrophysiological or microspectrophotometrical assessments of cone photoreceptors *ex vivo*, or absorbance spectroscopy of purified cone pigments *in vitro*, would be a valuable complement to the current dataset in refining our estimate for cone opsin spectral sensitivity. In particular, while our data clearly show that SWS opsin sensitivity peaks in the near-UV, the UV light filtering properties of the *R. pumilio* lens make it challenging to precisely define its λ_max_ based upon *in vivo* physiological responses. Unfortunately, our attempts to overcome this by recording retinal ganglion cell activity *ex vivo* using a multielectrode array were hindered by the thick inner limiting membrane of *R. pumilio*. Our best estimate is that the SWS opsin λ_max_ lies around 360 nm. First, the ERG revealed photopic spectral sensitivity that was best described by the weighted sum of two nomograms, with λ_max_ of 360 and ∼500 nm, and ratio of ∼1:4. Second, we assessed the spectral sensitivity in the visual thalamus of anaesthetised *R. pumilio*, and responses of individual neurons could be well described by single opsin spectral sensitivity estimates: approximately 75% of neurons with λ_max_ of ∼500 nm, and the remaining neurons with λ_max_ of <400 nm (tested wavelengths did not provide reliable discrimination at shorter wavelengths but results are fully compatible with λ_max_=360 nm). Likewise, across the population of dLGN neurons we recorded, overall spectral sensitivity could be very well explained by a combination of two pigments with λ_max_ of 360 and 501 nm. Lastly, we were able to validate these putative λ_max_ values by applying the technique of receptor silent substitution. In this case, a pair of spectrally distinct stimuli, designed to be isoluminant for pigments with λ_max_ of 360 nm and 500 nm, evoked no measurable ERG response.

We also explored the spectral sensitivity of *R. pumilio* vision in the dLGN under conditions of dark adaptation. These data indicated a λ_max_ of ∼493 nm, which is typical for rod vision in terrestrial mammals (classically ∼500 nm). However, light adaptation and/or a change in temporal frequency to bias responses towards cones produced only a small shift in spectral sensitivity, with MWS cones having a λ_max_ shifted approximately 7 nm towards longer wavelength. This similarity indicates that there is a limited adjustment in the spectral sensitivity of *R. pumilio* vision across the day–night cycle – or a low-amplitude Purkinje shift.

While the presumed spectral sensitivity of *R. pumilio* cone (and rod) opsins is remarkably similar to those of its close nocturnal relatives, the filtering properties of the *R. pumilio* lens attenuates the amount of short wavelength light actually reaching the *R. pumilio* retina. To put this into context, the *R. pumilio* lens attenuates the activation of the SWS cone to natural daylight by ∼70% (compared with the mouse lens which is close to ∼15%). While a long-pass property is a common feature of lenses in diurnal animals, it is most commonly paired with a concurrent shift in opsin spectral sensitivity towards longer wavelengths. To our knowledge, a combination of <400 nm SWS opsin λ_max_ and UV-filtering lens is highly unusual within the animal kingdom (Ellingson et al., 1995). This pairing, in effect, means that SWS cone sensitivity is dramatically curtailed at shorter wavelengths to leave it responsive to only a narrow portion of the spectrum. But despite this narrowing in sensitivity, we found evidence that the *R. pumilio* SWS cone contributes to activity throughout the visual projection. The utility of this more narrow-band UV-sensitive pigment in *R. pumilio*, however, remains a matter of speculation. The most parsimonious explanation is that this combination is a compromise, on the one hand restricting the amount of UV light reaching the *R. pumilio* retina (to protect the retina from damage and/or enhance acuity) and on the other, retaining enough ‘functional’ UV sensitivity for a particular purpose. One possible reason for this UV sensitivity could be to allow violet–green colour discrimination (e.g. Joesch and Meister, 2016). The presence of separate cone classes expressing SWS and MWS opsins supports this notion*.* We have not explicitly tested that hypothesis in the current study, though it is notable that SWS- and MWS-evoked responses appear to remain separate at least at the level of the visual thalamus, implying that chromatic discrimination would be available to higher level visual processing. An alternative function could relate to evidence that shorter wavelength sensitivity can enhance contrast detection for light coming from the sky (Baden et al., 2013), which could improve detection of overhead predators.

Our findings have implications for using *R. pumilio* as a laboratory organism. Diurnal rodents could be useful alternatives to non-human primates and companion-animal species for examining cone/photopic vision. *Rhabdomys pumilio* can be maintained as a breeding colony in standard rodent facilities (provided that they receive appropriate environmental enrichment) and are reliably diurnal in both the laboratory and wild. This first description of their visual physiology confirms the presence of adaptations to a diurnal niche in their visual system and their potential for studies of cone-based vision.
